# An Analysis of the Influence of a Patient’s Sex on Quality of Life in Liver and Kidney Transplantation

**DOI:** 10.3390/healthcare12212116

**Published:** 2024-10-23

**Authors:** Naiara Campillo Amo, Enrique Pérez Martínez, Ana van-der Hofstadt Gomis, Ana Carolina Londoño Ramírez, Carlos J. van-der Hofstadt Román

**Affiliations:** 1 Departamento de Medicina Clínica, Universidad Miguel Hernández (UMH), 03550 Alicante, Spain; naiarajv2@gmail.com (N.C.A.); eperezmar@yahoo.es (E.P.M.); 2 Servicio de Psiquiatría, Hospital General Universitario Dr. Balmis, 03010 Alicante, Spain; 3 Instituto de Investigación Sanitaria y Biomédica de Alicante (ISABIAL), 03010 Alicante, Spain; cjvander@umh.es; 4 Departamento de Psicología de la Salud, Universidad Miguel Hernández (UMH), 03202 Alicante, Spain; 5 Departamento de Farmacología, Pediatría y Química Orgánica, Universidad Miguel Hernández (UMH), 03550 Alicante, Spain; 6 Servicio de Farmacología Clínica, Hospital General Universitario Dr. Balmis, 03010 Alicante, Spain; 7 Unidad de Psicología Hospitalaria, Hospital General Universitario Dr. Balmis, 03010 Alicante, Spain

**Keywords:** liver transplantation, kidney transplantation, quality of life, gender differences, gender perspective

## Abstract

Background: Renal and liver transplantation influences the quality of life of the patients who undergo these procedures. Therefore, the aim of the present study was to analyze possible differences in liver and kidney transplantation in relation to the patient’s sex and to determine their impact on quality of life. Methodology: An observational study was carried out with 147 patients with liver (*n* = 70) and kidney (*n* = 77) failure on the transplantation waiting list. The possible influence of sex on clinical, sociodemographic, and psychological aspects of the patients’ quality of life before and 6 months after transplantation was analyzed. Questionnaires on health-related quality of life (SF-36), the perception of social and family support (EASP), and coping strategies (CEA), the depression and anxiety scale (HAD), and the Eysenck personality inventory (EPI) were used. A univariate analysis was performed according to sex using statistical tools including the Chi-square test, the t-test, and a univariate linear analysis of variance. Results: In patients on the waiting list for liver transplantation, we found sex differences in terms of age (*p* = 0.040), time of evolution of end-stage liver disease (*p* = 0.013), etiology (*p* = 0.07), and associated complications, as well as in the consumption of tobacco and other psychotropic substances (*p* = 0.022), while patients on the waiting list for renal transplantation showed sex-related differences in terms of etiology (*p* = 0.012) and alcohol consumption (*p* = 0.005). The results showed significant sex-related differences in sociodemographic and psychological aspects, but no significant sex-related differences were observed in global quality of life in either of the two assessments in both groups. Discussion: The findings suggest that improvement in quality of life after liver or kidney transplantation is not influenced by the patient’s sex.

## 1. Introduction

In the last two decades, a greater emphasis has been placed on biological and health differences between the sexes with respect to diagnostic and therapeutic responses, especially with regard to mortality and morbidity [1]. A recent exhaustive review showed that sex and gender are important risk factors in practically every disease [2]. In this regard, a higher incidence of life-threatening diseases, mainly cardiovascular, has been observed in men. Women, on the other hand, have a higher prevalence of chronic disabling disorders, such as autoimmune diseases, and a higher propensity to experience depressive and anxiety disorders [3,4]. As for liver and kidney transplantation, many studies have mainly focused on biomedical factors, such as the characteristics of the donor, the survival rates, the causes of the disease, or the symptoms and their severity. Sex, as a variable, can influence other conditions, not only hormonal and immunological but also psychological and socioeconomic ones, which affect the health and quality of life of this population [5,6]. The disparity in different areas of life as a function of sex and gender, such as economic status, employment, income, access to medical care, and clinical results, can also be expanded to the area of transplantations [4,7,8].

It is important to differentiate between the terms “sex” and “gender”. “Sex” refers to the biological and physiological characteristics of an individual, while “gender” refers to the roles and behaviors that are socially constructed by men and women, which are influenced by sociodemographic factors [9].

The previously published literature has described differences with respect to the general survival rates in these types of transplantations in which men, due to a greater association with cardiovascular diseases, have a worse prognosis [9]. Likewise, a proportional increase in the general risk of death has been observed, specifically in the male sex, with respect to aging [10,11]. Nevertheless, when the quality of life assessment was included, it was found to be negatively associated with female gender [10]. Other psychosocial aspects have also been evaluated according to sex and gender, such as lower socio-familial support and lower income in women, a higher risk of transplantation, more concerns associated with the desire to become pregnant, as well as hormonal and immunological factors, etc. [6,10,12]. Similarly, no psychosocial factors have been found to be negatively associated with the quality of life of the male sex, although there are the protective effects of a higher education, active employment, and being married [10,13].

Furthermore, studies on the quality of life variable have shown that it has a great impact on the development, treatment, and prognosis of diseases. Its study and early approach have been related to obtaining benefits in the overall course of pathologies, and it is one of the main objectives of solid organ transplantation, along with the increase in the survival rates of the recipients, the graft, and the decrease in the morbidity rates [5]. More specifically, in the area of kidney transplantations, many studies coincide in that there is a significant improvement after a transplantation as compared to a dialysis, with improvements of around 80% in physical, mental, and psychological activities and 60% with respect to social aspects [14,15]. In the case of a liver transplantation, results suggest that more than 80% of the patients are able to recover their normal activities [15]. Likewise, the initial study on which this sub-analysis was performed was able to corroborate that the post-transplantation quality of life was greater than before the transplantation. It was also found that aspects such as high neuroticism, anxiety, and depression reduced the quality of life scores of the patients who underwent the transplantation [16].

A recent study on the influence of gender on quality of life in transplant patients reflected low scores for perceived quality of life and higher risks of hospitalization, graft failure, and mortality [17], and a significant relationship was found with female sex [13]. Other studies have demonstrated that female sex, advanced age, a lower level of education, being single, and being unemployed implied negative associations with the quality of life of patients with a kidney transplantation [14]. In kidney transplantations, the reviews that were found showed a low representation of the female sex in worldwide studies, as well as difficulties in accessing transplantation lists and a lower probability of completing all the health care stages after the transplantation. This raises the possibility of an influence of non-biological factors on the health care processes of these patients [9].

The analysis of the co-variables that influence the quality of life in liver and kidney transplantations from a sex and gender perspective can help to identify psychosocial and demographic patterns associated with the etiology of the underlying pathology. This could be useful for assessing the states of vulnerability and possible strategies of prevention and treatment that are more effective.

For this reason, the primary objective of the present work was to analyze the possible differences in liver and kidney transplantation with respect to sex and to determine the influence of sex on quality of life. Likewise, the intention was to identify any differences in influence between men and women in terms of the sociodemographic, clinical, and psychological variables.

## 2. Materials and Methods

In the present study, an anonymized database of patients on the waiting list (*n* = 147) for a liver (*n* = 70) or kidney (*n* = 77) transplantation was used. The database was derived from a previous study conducted between 2013 and 2015 at the General University Hospital Dr. Balmis of Alicante [16]. This study examined the influence of personality characteristics on the quality of life of liver and kidney pre-transplantation and post-transplantation patients and was approved by the Ethics Committee of Clinical Research at the General University Hospital Dr. Balmis (Ref. CEIC PI2013/28).

The sample included all the older patients who had been admitted and remained in the pre-transplantation programs during the study period. Likewise, it was necessary for the patients to have provided their informed consent to participate in the study, to have the capacity and autonomy to complete the self-applied tests, and to answer more than 95% of the questions in the questionnaires.

Of the 147 patients on the waiting list, 79 were subjected to a transplantation and were monitored for 6 months during the period between June 2013 and July 2014 (42 received a kidney transplantation and 37 a liver transplantation). Data were collected when the patient was included on the waiting list for the liver or kidney transplantation, as well as 6 months after the interventions. The baseline sample was analyzed to assess the influence of sex in the 147 patients included, and the follow-up analysis was performed with the 79 transplanted patients.

Primary outcomes:

− The quality of life variables were analyzed using the validated Spanish version of the SF-36 questionnaire on health-related quality of life [18,19].

Secondary outcomes:

− Sociodemographic variables: Sociodemographic variables such as sex, age, marital status, level of education, type of occupation, and level of income.
− Clinical variables: Etiology of the disease, age at diagnosis, length of time with the illness and deterioration, hospitalizations in the last year and causes, days at the hospital, packs of tobacco consumed per year, consumption of other psychotropic drugs, years of alcohol consumption, and standard drink units per day. Sociodemographic and clinical variables were recorded in the data research collection of the previous study.
− Neuroticism/extroversion: This variable was collected using the validated Spanish version of Eysenck’s Personality Inventory (EPI) [20,21], showing a good internal consistency.
− Anxiety and depression disorders: This variable was collected using the validated Spanish version of the Hospital Anxiety and Depression (HAD) scale [22,23].
− Socio-family support: This variable was evaluated through the use of the Perception of Social and Family Support Scale (EASP) [24].
− Coping strategies: This variable was evaluated using the Coping Strategies Questionnaire (CEA) [25].

With respect to the data analysis, SPSS version 25.0 statistical software was utilized (SPSS Inc., Chicago, IL, USA). The hypothesis contrast was two-tailed, with a significance of 0.05 and a 95% confidence interval. The categorical variables are described as frequencies and percentages, and the continuous variables are described as means and standard deviations (SDs). A univariate analysis was performed for the description of the sample according to sex. The differences between the categorical variables were determined using the Chi-square test, and the analysis of the continuous variables was performed with the t-test for independent samples.

A one-way analysis of variance (ANOVA) was performed to analyze the differences in the quality of life as a function of sex, study period, and type of transplantation; their interactions were analyzed through a multivariate analysis.

## 3. Results

In the present study, a total of 147 patients were included on the liver and kidney transplantation list (*n* = 70 and *n* = 77, respectively). Among the two groups, a lower number of women was observed in the liver group (*n* = 12) than in the kidney group (*n* = 31). Given that this difference was statistically significant (*p* = 0.021), a separate analysis was performed of both groups as a function of sex and its distribution through time (pre-transplantation and post-transplantation).

Table 1 describes characteristics of patients on the waiting list for a liver transplantation.

### 3.1. Sociodemographic Characteristics for Liver Patients

The marital status, level of education, occupation, level of income, mean age, and age at diagnosis of the patients on the liver transplantation waiting list were studied (review Supplementary Materials).

Significant differences were found with respect to age between men (54.57 years) and women (60.75 years) (*p* = 0.040) (Table 1). With respect to the remaining characteristics studied, no statistically significant differences were found.

### 3.2. Clinical Characteristics for Liver Patients

We found statistically significant differences in many variables among the clinical characteristics, as shown in Table 1. With respect to the length of time with the illness (*p* = 0.013), it was observed that the mean for the men (8.31) was lower than that for the females (15.33). Statistically significant differences were also found with respect to the etiology of the end-stage liver disease (*p* = 0.007), with alcohol-related cirrhosis (men: 70.2%; women: 36.4%) and viral cirrhosis (men: 17.5%; women: 36.4%) being the most common causes in both sexes. With respect to the cause of hospitalization (*p* = 0.025), infections (19.1%) and encephalopathies (19.1%) were underlined in men and ascites (50%) and encephalopathy (20%) in the case of women. Significant differences were also found with respect to tobacco consumption, with 81% of men and 50% of women being smokers (*p* = 0.022), and other psychotropic drugs (*p* = 0.020), with 32.8% of men and 0% of women being consumers of these substances.

### 3.3. Psychological Characteristics for Liver Patients

Statistically significant differences were found (*p* = 0.027) with respect to the self-blame resource in the coping strategies, which was recorded in 74.1% of men and 41.7% of women. No significant differences were observed between sexes in the rest of the psychological variables analyzed (Table 1).

### 3.4. Quality of Life for Liver Patients

Differences between sexes in the SF-36 questionnaire on quality of life were analyzed (Table 2), with significantly higher mean scores found in men in terms of a physical limitation in activity (42.24; *p* = 0.048) and body pain (47.05; *p* < 0.001). However, in the rest of the items analyzed, the results were homogeneous between the sexes.

Description of patients on the waiting list for kidney transplantation:

### 3.5. Sociodemographic Characteristics for Kidney Patients

No significant differences between the sexes were found in the sociodemographic variables (Table 3) (review Supplementary Materials).

### 3.6. Clinical Characteristics for Kidney Patients

Statistically significant differences were found in the variables of etiology of the end-stage renal disease (*p* = 0.012), in the number of years of alcohol consumption (*p* = 0.005), and in the daily standard drink units (*p* = 0.001).

In relation to etiology, in the case of men, 37% presented GMN and 21.7% presented HTA, these being the two variables with the highest responses. In the case of women, 38.7% presented polycystic disease and 19.4% presented GMN.

Differences found in the consumption of alcohol were observed in both years of consumption (21.43 years in men and 6 years in women, *p* = 0.005) as well as the mean consumption in standard drink units (SDUs) (31.02 in men and 7.13 in women, *p* = 0.001) (Table 3). The remaining characteristics studied did not show statistically significant differences between men and women.

### 3.7. Psychological Characteristics for Kidney Patients

Significant differences were found in the use of religion as a coping strategy, with this characteristic observed in 13% of men as compared to 74.2% of women (*p* < 0.001). No statistically significant differences were observed in the rest of the psychological variables studied (Table 3).

### 3.8. Quality of Life for Kidney Patients

The quality of life before the kidney transplantation was assessed with the SF-36 questionnaire. No significant differences were found between sexes in any of the items studied (Table 4).

### 3.9. Post-Transplantation Study

The change in quality of life as a function of sex was analyzed in the patients who received a transplantation during the study period, utilizing the means reported at baseline and 6 months after the transplantation.

Significant differences were observed as a function of sex in the perception of body pain in the patients in the liver transplantation group (*p* = 0.011), considering that the average score increased over time in both sexes. Differences were also found in social function (*p* = 0.006), again showing how the mean of both groups increased over time.

On the other hand, differences were observed in the perception of general health (*p* = <0.001) and vitality (*p* = 0.025) as a function of time (Table 5), given that the means in both groups increased over time, which could suggest a higher quality of life on the part of the subjects.

The multivariate analysis of the quality of life items associated with sex and time for the liver transplantation group was controlled according to age, length of time being ill, etiology of the disease, cause of hospitalization, alcohol consumption, and smoking. These did not show significant differences.

On the other hand, in the patients in the kidney transplantation group, statistically significant differences were only observed as a function of time for the physical limitation (*p* = 0.006) and social function (*p* = 0.004), with the average for men decreasing and the average for women increasing. Statistical differences were also found for the general perception of health (*p* = 0.014) and the physical health summary (*p* = 0.047), evidenced by the mean in both variables increasing in both groups over time (Table 6).

The multivariate analysis of the items of quality of life associated with sex and time of the kidney transplantation group was controlled by the etiology of the disease and alcohol consumption. These did not show significant differences.

## 4. Discussion

The aim of this study was to analyze the possible differences in liver and kidney transplants with respect to sex and to determine their impact on quality of life. Another objective was to identify the influence of sociodemographic, clinical, and psychological variables between men and women.

In general terms, the analysis according to sex showed some differences in quality of life between men and women in liver and kidney transplantation.

In relation to the quality of life before liver transplantation, statistically significant differences were found in the means of physical limitation (*p* = 0.048) and body pain (*p* < 0.001), with women having lower scores than men. However, no statistically significant differences were found in the quality of life of the patients before kidney transplantation.

In terms of sex and time differences in the quality of life of patients who received a liver transplant, statistically significant sex differences were found in bodily pain (*p* = 0.011) and social functions (*p* = 0.006), with subject scores rising 6 months after the intervention. On the other hand, in terms of differences over time, statistically significant differences were found in general health perception (*p* < 0.001) and vitality (*p* = 0.025), with means increasing in both groups over time.

Regarding the differences associated with sex and time in the quality of life of patients who received a kidney transplant, statistically significant differences over time were found in physical limitation (*p* = 0.006) and social function (*p* = 0.004), with men and women exhibiting a lower and higher mean score after 6 months, respectively. Similarly, statistically significant differences were found in general health perception (*p* = 0.014) and physical health summary (*p* = 0.047), with both groups’ mean scores increasing at 6 months.

In this sense, both types of transplantations showed similar scores in patients on both waiting lists and in those who had undergone a transplantation 6 months after the intervention. These findings contradict those found in the literature reviewed, which reported significant negative associations between quality of life, social disadvantages, and the female sex/gender [10,14], even in terms of mortality [13].

In relation to the sociodemographic variables, statistically significant differences were found in the age of the patients on the waiting list for liver transplantation based on sex, with men having a lower average age (54.57) than women (60.75) (*p* = 0.040). Moreover, no influence of the socioeconomic factors of both sexes on the baseline quality of life results was observed, and no conclusive data were found in the current literature. Some studies indicate that male liver transplantation patients with a higher level of education experience a higher quality of life [26]. Likewise, another study with kidney transplantation patients showed better results in all the dimensions of physical and mental health in the male population, with a positive relationship with the level of education [27]. However, as found in our results, other studies did not find a statistically significant correlation between quality of life and sociodemographic factors such as age, gender [5,28], employment, education, or marital status [28].

A bias in the interpretation of these results could be the lack of homogeneity in the methodology, as these studies employed different quality of life questionnaires and were performed at different periods of time and with varying populations, which makes a direct comparison with them difficult. It is also possible that these contradicting results are influenced by cultural factors associated with sex. In this sense, it is necessary to continue to make advances in research in the field of transplantations using common strategies and questionnaires to establish correlations and define possible causes of discrepancies and to create new strategies to improve the quality of life of patients subjected to liver and kidney transplantations.

Differences were observed in the months with the disease (*p* = 0.013) in relation to the sex of the patients before liver transplantation, with women presenting a higher mean than men. Differences were also observed in terms of etiology (*p* = 0.007), with alcohol and viral infection being the most common causes in men and women, as well as in the causes of hospitalization (*p* = 0.025). Likewise, differences in etiology (*p* = 0.012) were observed in relation to the sex of patients prior to kidney transplantation, with GMN being the most common cause in men and polycystic disease being the most common in women.

Furthermore, significant differences were found in the consumption of toxic substances between men and women in both types of transplantation (*p* < 0.05). In the case of liver transplantation, a significantly greater consumption of tobacco and other psychotropic substances was observed in men, which was expected, as this pathology has an alcohol etiology in 70% of cases. This relationship was also observed in kidney transplantation, for which a higher consumption of alcohol was observed in men in terms of years of consumption and daily standard drink units. These results are consistent with the data obtained for the Spanish population and analyzed by the Spanish National Statistics Institute, which indicated a higher consumption of alcohol, tobacco, and other psychotropic substances in the male population [29].

No significant differences were observed between the sexes in the analysis of the global summaries of physical and mental health of the patients compared according to time. The findings, with respect to the improvement in both types of transplantations, are in agreement with the published literature [14,15]. However, higher physical limitation and perception of pain was found in women 6 months after liver transplantation. At present, the scientific literature provides diverse studies on the differences in the perception of pain between sexes. These studies have shown that this perception can be influenced by multiple factors, among them hormonal, behavioral, and sociocultural ones, coping strategies, and post-transplantation “fatigue” [30]. More specifically, a study with liver transplantation patients found a worse global score in women in the Psychosocial Adjustment to Illness Scale (PAIS) and a higher psychological dysfunction [31]. The results of the present work are in agreement with the trends in existing studies on patients with a liver transplantation, where the men reported significantly higher scores in the perception of pain, function, and physical limitation, while women recorded better scores with respect to emotional functions and mental health [30].

Although, on the other hand, few differences were observed between sexes with respect to the prevalence of mood disorders, personality characteristics, and the perception of support, which could influence the quality of life in both types of transplantations. The study of these co-variables is considered important in liver and kidney transplantations. Likewise, previous studies have shown that depression symptoms before a liver transplantation are associated with a significant increase, up to three or four times, in the risk of graft rejection and mortality 18 months after the procedure, though this relationship is not associated with sex or other socioeconomic factors [11]. In other studies, differences were found in the female sex with respect to depression in liver transplantation recipients, with an association observed with other confounding factors, such as advanced age, low economic level, and a higher number of associated comorbidities [32]. The results of a prospective cohort study with liver or kidney transplantation patients indicated that a depression symptomatology was more frequently reported in young women who live alone, smokers, those with lower levels of education, and those who took part in leisure activities less frequently [11].

The study of quality of life as a primary objective includes the study of the expectations of transplanted patients and, overall, the detection of important components of action to address the gender inequalities involved. More specifically, an experimental pilot study examined an intervention in the form of group therapy to work on the coping skills in a candidate population for a liver or kidney transplantation, with the intention to decrease anxiety and depression. Statistically significant positive results that persisted through time were obtained, without differences between sexes in the results before or after the intervention or during the follow-up. Psychological and psychiatric support and therapeutic intervention can be additional tools for the acceptance of the state of health, the establishment of future objectives, and active integration in society. Nevertheless, the results of the present study on the use of coping strategies were similar between sexes, with only a higher occurrence of self-blame observed in the liver transplantation male population, perhaps associated with a higher percentage of alcohol cirrhosis etiology and religiousness in kidney transplantations in the female population [33].

Our results highlight the relevance of considering sex in the assessment of quality of life and the identification of vulnerability factors, as well as socioeconomic and psychological ones, which may contribute to the deterioration of the health of patients subjected to both types of transplantations. This leads us to propose future improvements in health care programs and the development of personalized health care strategies based on gender [9].

As for the limitations of the present study, it is important to underline that the population included in the study was part of the Spanish Public Health System, with this system influencing the results that were obtained, which does not allow us to compare these results with other results in other health systems. This could explain some of the differences found with the literature with respect to the relationship between quality of life and socioeconomic factors. Likewise, the concept of gender present in the society studied also has an influence, as it is understood as a construct of an individual within a society. The study also did not have a large sample due to the specificity of the characteristics. Nevertheless, we must point out the complexity in obtaining such a number of transplanted patients.

As this was a retrospective study, the results did not encompass the social constructs, the influence of roles, the self-perception of the patients, or their social behavior and interaction. Although the variable sex was used from a perspective of gender, and other known sociodemographic variables were used, the study did not use specific variables to measure the impact of the dimensions of gender on the quality of life of the patients. This limits the possibility of obtaining a more detailed view of the gender differences in the patients on the waiting lists for liver and kidney transplantations. As a result, future studies could include variables that allow for a more complete assessment of the effect of these dimensions on the quality of life of the patients on the liver or kidney transplantation waiting list [34].

After analyzing the data obtained in the study, the following conclusions can be drawn:

(a) Quality of life is an essential marker in the study of liver and kidney transplantations, and its combination with sociodemographic, clinical, and psychological factors can be useful in the identification of specific intervention areas according to the sex and gender of the patient.
(b) No significant differences were found in the overall indices of physical and mental quality of life between sexes in the context of liver and kidney transplants, except in the case of pain in liver transplantation. The presence of greater physical limitation and social function pre-transplant and the significant difference in the perception of pain in women points to the need for strategies to improve quality of life.
(c) The understanding of the impact of gender on the quality of life of transplanted patients is limited by cultural differences and a lack of studies with a gender perspective. Therefore, more studies must be conducted in this area to enable more effective and personalized health care.

## Figures and Tables

**Table 1:** Description of the sociodemographic, clinical, and psychological characteristics of patients on the waiting list for liver transplantation based on sex.

Variables	Men (*n* = 58)	Women (*n* = 12)	*p*-Value
Sociodemographic characteristics			
Age (years); mean (SD)	54.57 (8.21)	60.75 (4.55)	0.040 **
Age at diagnosis (years); mean (SD)	46.26 (10.48)	45.42 (13.95)	0.322
Clinical features/use of psychotropic drugs			
Length of time with illness (months); mean (SD)	8.31 (8.60)	15.33 (13.70)	0.013 **
Etiology; *n* (%)			
Alcohol	40 (70.2%)	4 (36.4%)	0.007 **
Viral	10 (17.5%)	4 (36.4%)	
Polycystic disease	0 (0%)	2 (18.2%)	
Cancer	5 (8.8%)	1 (9.1%)	
Causes of hospitalization; *n* (%)			
Infection	9 (19.1%)	1 (10%)	0.025 **
Encephalopathy	9 (19.1%)	2 (20%)	
Esophageal varices	8 (17%)	0 (0%)	
Ascites	5 (10.6%)	5 (50%)	
Alcohol consumption (years); mean (SD)	32.67 (32.77)	8.33 (12.70)	0.807
SDU (g/day); mean (DS)	126.66 (122.16)	32.92 (91.16)	0.065
Smoker; *n* (%)	47 (81%)	6 (50%)	0.022 **
Other psychotropic drugs; *n* (%)	19 (32.8%)	0 (0%)	0.020 **
Psychological characteristics			
Anxiety disorder; *n* (%)	11 (19%)	3 (25%)	0.634
Depressive disorder; *n* (%)	12 (20.7%)	5 (41.7%)	0.123
Neuroticism; *n* (%)			
Low	26 (44.8%)	2 (16.7%)	0.101
Medium	8 (13.8%)	1 (8.3%)	
High	24 (41.4%)	9 (75%)	
Extroversion; *n* (%)			
Low	20 (34.5%)	6 (50%)	0.509
Medium	11 (19%)	1 (8.1%)	
High	27 (46.6%)	5 (41.7%)	
Number of providers; mean (SD)	5.52 (2.11)	4.67 (2.06)	0.980
Satisfaction with support; mean (SD)	4.30 (0.59)	4.44 (0.47)	0.346
Level of support; mean (SD)	23.60 (9.72)	20.58 (8.49)	0.718
Positive thinking; *n* (%)	56 (96.6%)	12 (100%)	0.514
Searching for advantages; *n* (%)	51 (87.9%)	11 (91.7%)	0.711
Resignation; *n* (%)	46 (79.3%)	12 (100%)	0.083
Searching for support; *n* (%)	41 (70.7%)	11 (91.7%)	0.130
Searching for solutions; *n* (%)	40 (69%)	9 (75%)	0.678
Wishful thinking; *n* (%)	45 (77.6%)	12 (100%)	0.069
Repress emotions; *n* (%)	44 (75.9%)	8 (66.7%)	0.507
Self-blame; *n* (%)	43 (74.1%)	5 (41.7%)	0.027 **
Religiousness; *n* (%)	27 (46.6%)	9 (75%)	0.073
Escapism; *n* (%)	32 (55.2%)	4 (33.3%)	0.168
Blaming others; *n* (%)	18 (31%)	4 (33.3%)	0.876

Note: ** *p* < 0.05 (Cramer’s V = 0.02); SD = standard deviation; SDU = standard drink unit.

**Table 2:** Table of results of the quality of life questionnaire (SF-36), broken down by sex, in the population described before liver transplantation.

Quality of Life Items (SF-36)	Men (*n* = 58)	Women (*n* = 12)	*p*-Value
Physical function; mean (SD)	65.23 (23.70)	42.08 (26.58)	0.651
Physical limitation; mean (SD)	42.24 (42.45)	16.67 (38.92)	0.048 **
Body pain; mean (SD)	47.05 (14.49)	31.58 (31.01)	<0.001 **
General perception of health; mean (SD)	43.38 (20.54)	38.33 (20.44)	0.918
Vitality; mean (SD)	46.21 (30.42)	31.67 (27.47)	0.853
Social function; mean (SD)	71.01 (28.11)	38.54 (33.05)	0.660
Mental health; mean (SD)	65.12 (26.89)	57.67 (21.06)	0.387
Emotional limitation; mean (SD)	78.97 (37.08)	61.08 (44.61)	0.142
Physical health summary; mean (SD)	36.80 (9.49)	32.87 (13.85)	0.104
Mental health summary; mean (SD)	46.46 (11,75)	37.25 (13.03)	0.398

Note: ** *p* < 0.05 (Cramer’s V = 0.02); SD = standard deviation.

**Table 3:** Description of the sociodemographic, clinical, and psychological characteristics of patients on the waiting list for kidney transplantation based on sex.

Variables	Men (*n* = 46)	Women (*n* = 31)	*p*-Value
Sociodemographic characteristics			
Age (years); mean (SD)	51.28 (12.45)	54 (12.25)	0.384
Age at diagnosis (years); mean (SD)	41.22 (17.22)	39.97 (16.38)	0.753
Clinical features/use of psychotropic drugs			
Length of time with illness (months); mean (SD)	12.24 (10.37)	14.03 (10.28)	0.648
Etiology; *n* (%)			
GMN	17 (37%)	6 (19.4%)	0.012 **
HTA	10 (21.7%)	2 (6.5%)	
Polycystic disease	3 (6.5%)	12 (38.7%)	
DM	5 (10.9%)	4 (12.9%)	
Causes of hospitalization; *n* (%)			
Infection	10 (34.5%)	5 (29.4%)	0.610
Surgery	9 (31%)	7 (41.2%)	
Alt.FR	3 (10.3%)	2 (11.8%)	
HTA/DM	2 (6.9%)	3 (17.6%)	
Dialysis; *n* (%)	34 (73.9%)	28 (90.3%)	0.075
Alcohol consumption (years); mean (SD)	21.43 (17.93)	6 (13.17)	0.005 **
SDUs (g/day); mean (SD)	31.02 (34.20)	7.13 (19.52)	0.001 **
Smoker; *n* (%)	33 (71.7%)	19 (61.3%)	0.337
Other psychotropic drugs; *n* (%)	4 (8.7%)	0 (0%)	0.092
Psychological characteristics			
Anxiety disorder; *n* (%)	8 (17.4%)	7 (22.6%)	0.573
Depressive disorder; *n* (%)	3 (6.5%)	5 (16.1%)	0.175
Neuroticism; *n* (%)			
Low	28 (60.9%)	17 (54.8%)	0.567
Medium	5 (10.9%)	2 (6.5%)	
High	13 (28.3%)	12 (38.7%)	
Extroversion; *n* (%)			
Low	14 (30.4%)	6 (19.4%)	0.503
Medium	5 (10.9%)	5 (16.1%)	
High	27 (58.7%)	20 (64.5%)	
Number of providers; mean (SD)	5.28 (2.05)	5.84 (1.71)	0.318
Satisfaction with support; mean (SD)	4.33 (0.62)	4.21 (0.48)	0.492
Level of support; mean (SD)	22.78 (9.41)	24.68 (7.94)	0.359
Positive thinking; *n* (%)	46 (100%)	29 (93.5%)	0.081
Searching for advantages; *n* (%)	43 (93.5%)	31 (100%)	0.147
Resignation; *n* (%)	33 (71.7%)	26 (83.9%)	0.217
Searching for support; *n* (%)	34 (73.9%)	25 (80.6%)	0.494
Searching for solutions; *n* (%)	34 (73.9%)	24 (77.4%)	0.726
Wishful thinking; *n* (%)	28 (60.9%)	22 (71%)	0.362
Repress emotions; *n* (%)	39 (84.8%)	23 (74.2%)	0.250
Self-blame; *n* (%)	10 (21.7%)	5 (16.1%)	0.542
Religiousness; *n* (%)	6 (13%)	23 (74.2%)	<0.001 **
Escapism; *n* (%)	15 (32.6%)	13 (41.9%)	0.404
Blaming others; *n* (%)	9 (19.6%)	5 (16.1%)	0.701

Note: ** *p* < 0.05 (Cramer’s V = 0.02); SD = standard deviation; SDU = standard drink unit; GMN = glomerulonephritis; HTA = hypertension; DM = diabetes mellitus; Alt.FR = alterations in renal function.

**Table 4:** Table of results of the quality of life questionnaire (SF-36), broken down by sex, in the population described before kidney transplantation.

Quality of Life Items (SF-36)	Men (*n* = 46)	Women (*n* = 31)	*p*-Value
Physical function; mean (SD)	72.27 (21.41)	67.50 (20.64)	0.640
Physical limitation; mean (SD)	54.89 (44.29)	52.42 (42.00)	0.312
Body pain; mean (SD)	54.93 (20.17)	55.06 (19.41)	0.475
General perception of health; mean (SD)	53.63 (20.36)	50.52 (19.66)	0.831
Vitality; mean (SD)	58.15 (21.94)	54.68 (27.44)	0.169
Social function; mean (SD)	80.16 (24.51)	77.01 (25.43)	0.735
Mental health; mean (SD)	76.70 (17.22)	67.58 (25.18)	0.068
Emotional limitation; mean (SD)	90.59 (26.91)	84.97 (34.24)	0.132
Physical health summary; mean (SD)	40.01 (8.83)	39.82 (7.34)	0.105
Mental health summary; mean (SD)	51.89 (9.28)	48.71 (12.82)	0.143

Note: SD = standard deviation.

**Table 5:** Table of results broken down by items of the quality of life questionnaire (SF-36), differentiated by sex and time (initial study and follow-up at 6 months), in patients who received liver transplantation.

Quality of Life Items (SF-36)	Baseline Measurements		Measurement After 6 Months		*p*-Value Sex	*p*-Value Time
	Men (*n* = 31)	Women (*n* = 6)	Men (*n* = 31)	Women (*n* = 6)		
Physical function; mean (SD)	67.19 (22.40)	50.83 (23.96)	71.93 (22.23)	70.00 (11.83)	0.127	0.254
Physical limitation; mean (SD)	45.96 (44.29)	16.67 (40.82)	49.45 (45.74)	18.00 (21.30)	0.127	0.534
Body pain; mean (SD)	46.22 (13.85)	28.00 (28.00)	55.35 (17.42)	43.33 (29.84)	0.011 **	0.099
General perception of health; mean (SD)	44.19 (19.00)	35.33 (22.86)	71.83 (18.82)	66.00 (32.58)	0.071	<0.001 **
Vitality; mean (SD)	51.29 (29.21)	32.50 (31.42)	63.06 (22.82)	60.83 (20.10)	0.195	0.025 **
Social function; media (DS)	71.61 (29.56)	31.33 (31.47)	80.00 (26.67)	64.83 (38.30)	0.006 **	0.070
Mental health; mean (SD)	61.93 (26.75)	63.33 (20.14)	74.96 (20.68)	62.66 (24.61)	0.344	0.401
Emotional limitation; mean (SD)	70.96 (41.07)	72.16 (32.92)	77.41 (38.87)	50.00 (45.99)	0.817	0.224
Physical health summary; mean (SD)	38.83 (8.28)	35.00 (16.29)	42.61 (7.40)	40.00 (3.68)	0.189	0.259
Mental health summary; mean (SD)	44.96 (12.77)	36.16 (8.56)	50.19 (11.26)	42.50 (16.62)	0.083	0.055

Note: ** *p* < 0.05 (Cramer’s V = 0.02); SD = standard deviation.

**Table 6:** Table of results broken down by items of the quality of life questionnaire (SF-36), differentiated by sex and time (initial study and control at 6 months), in patients who received kidney transplantation.

Quality of Life Items (SF-36)	Baseline Measurements		Measurement After 6 Months		*p*-Value Sex	*p*-Value Time
	Men (*n* = 26)	Women (*n* = 16)	Men (*n* = 26)	Women (*n* = 16)		
Physical function; mean (SD)	74.61 (17.82)	70.31 (16.57)	71.53 (19.01)	68.75 (20.12)	0.298	0.085
Physical limitation; mean (SD)	54.80 (44.73)	48.43 (40.27)	50.96 (47.68)	51.56 (45.15)	0.995	0.006 **
Body pain; mean (SD)	55.00 (20.99)	52.56 (17.36)	57.84 (22.25)	51.87 (20.61)	0.754	0.601
General perception of health; mean (SD)	56.38 (19.27)	51.18 (20.27)	65.53 (20.53)	66.62 (22.14)	0.483	0.014 **
Vitality; mean (SD)	56.34 (18.14)	56.25 (30.46)	63.07 (20.69)	52.50 (23.30)	0.200	0.592
Social function; media (DS)	82.30 (23.98)	74.50 (26.63)	79.07 (20.73)	76.00 (28.50)	0.400	0.004 **
Mental health; mean (SD)	77.84 (16.40)	65.18 (28.05)	76.30 (18.92)	76.00 (18.30)	0.128	0.606
Emotional limitation; mean (SD)	93.61 (21.08)	87.50 (34.15)	91.03 (24.14)	70.81 (45.35)	0.107	0.398
Physical health summary; mean (SD)	40.34 (8.36)	39.81 (8.24)	41.38 (9.45)	41.87 (9.45)	0.916	0.047 **
Mental health summary; mean (SD)	52.34 (8.95)	48.37 (13.38)	52.34 (9.05)	48.12 (11.87)	0.092	0.778

Note: ** *p* < 0.05 (Cramer’s V = 0.02); SD = standard deviation.

## Data Availability

The data are not publicly available due to ethical reasons.

## Supplementary Materials

The following supporting information can be downloaded at: https://www.mdpi.com/article/10.3390/healthcare12212116/s1, Table S1. Dates of socioeconomic status.

## References

[1] Lee S.K. (2018). Sex as an important biological variable in biomedical research. BMB Rep..

[2] Mauvais-Jarvis F., Merz N.B., Barnes P.J., Brinton R.D., Carrero J.-J., DeMeo D.L., De Vries G.J., Epperson C.N., Govindan R., Klein S.L. (2020). Sex and gender: Modifiers of health, disease, and medicine. Lancet.

[3] Wang H., Zhang X., Li H., Sun Z., Zhong Y. (2023). Gender differences in the burden of multiple sclerosis in China from 1990 to 2019 and its 25-year projection: An analysis of the Global Burden of Diseases Study. Health Sci. Rep..

[4] Cherepanov D., Palta M., Fryback D.G., Robert S.A., Hays R.D., Kaplan R.M. (2011). Gender Differences in Multiple Underlying Dimensions of Health-related Quality of Life Are Associated with Sociodemographic and Socioeconomic Status. Med. Care.

[5] Ordin Y.S., Dicle A., Wellard S. (2011). Quality of Life in Recipients before and after Liver Transplantation in Turkey. Prog. Transplant..

[6] Maenosono R. (2023). Sex difference and immunosenescence affect transplantation outcomes. Front. Transplant..

[7] Gordon E.J., Ladner D.P. (2012). Gender Inequities Pervade Organ Transplantation Access. Transplantation.

[8] Puoti F., Ricci A., Nanni-Costa A., Ricciardi W., Malorni W., Ortona E. (2016). Organ transplantation and gender differences: A paradigmatic example of intertwining between biological and sociocultural determinants. Biol. Sex Differ..

[9] Salas M.A.P., Chua E., Rossi A., Shah S., Katz-Greenberg G., Coscia L., Sawinski D., Adey D. (2022). Sex and gender disparity in kidney transplantation: Historical and future perspectives. Clin. Transplant..

[10] Alkatheri A., Al Bekairy A., Aburuz S., Qandil A., Khalidi N., Abdullah K., Al Sayyari S., Bustami R., Al Harbi S., Al Raddadi S. (2015). Exploring quality of life among renal and liver transplant recipients. Ann. Saudi Med..

[11] Corruble E., Barry C., Varescon I., Durrbach A., Samuel D., Lang P., Castaing D., Charpentier B., Falissard B. (2011). Report of depressive symptoms on waiting list and mortality after liver and kidney transplantation: A prospective cohort study. BMC Psychiatry.

[12] Melk A., Babitsch B., Borchert-Mörlins B., Claas F., Dipchand A.I., Eifert S., Eiz-Vesper B., Epping J., Falk C.S., Foster B. (2019). Equally Interchangeable? How Sex and Gender Affect Transplantation. Transplantation.

[13] Schneekloth T.D., Hitschfeld M.J., Petterson T.M., Narayanan P., Niazi S.K., Jowsey-Gregoire S.G., Thusius N.J., Vasquez A.R., Kremers W.K., Watt K.D. (2018). Psychosocial Risk Impacts Mortality in Women After Liver Transplantation. Psychosomatics.

[14] Sainz-Barriga M., Baccarani U., Scudeller L., Risaliti A., Toniutto P., Costa M., Ballestrieri M., Adani G., Lorenzin D., Bresadola V. (2005). Quality-of-Life Assessment Before and After Liver Transplantation. Transplant. Proc..

[15] Ruiz D.D., Aguirre C.F., González F.S., Ruiz M.C. (2008). Indicaciones y resultados a largo plazo de los trasplantes de órganos sólidos. Calidad de vida en pacientes trasplantados. Med. Intensiv..

[16] Londoño Ramírez A.C. (2017). Influencia de Los Patrones Psiquiátricos y Psicológicos Sobre la Calidad de Vida en el Trasplante Renal y Hepático [Tesis en Internet]. Alicante: Universidad Miguel Hernández de Elche. https://dspace.umh.es/bitstream/11000/4401/1/TD%20Londo%C3%B1o%20Ram%C3%ADrez%2C%20Ana%20Carolina.pdf.

[17] Tarabeih M., Bokek-Cohen Y., Azuri P. (2020). Health-related quality of life of transplant recipients: A comparison between lung, kidney, heart, and liver recipients. Qual. Life Res..

[18] Ware J.E., Kosinski M., Dewey J.E., Gandek B. (2000). SF-36 Health Survey: Manual and Interpretation Guide.

[19] Zigmond A.S., Snaith R.P. (1983). The Hospital Anxiety and Depression Scale. Acta Psychiatr. Scand..

[20] Alonso J., Prieto L., Antó J.M. (1995). La versión española del SF-36 Health Survey (Cuestionario de Salud SF-36): Un instrumento para la medida de los resultados clínicos. Med. Clin..

[21] Eysenk H.J., Eysenk S.B.G. (1964). Manual of the Eysenck Personality Inventory.

[22] Sánchez M., Cuadras C. (1972). Adaptación española del cuestionario E.P.I. de Eysenck. Anu. Psicol..

[23] Terol M.C., López-Roig S., Rodríguez-Marín J., Martí-Aragón M., Pastor M.A., Reig M.T. (2007). Propiedades psicométricas de la escala hospitalaria de ansiedad y depresión (HAD) en población española. Ansiedad Estrés.

[24] Terol M.C., López-Roig S., Pastor M.A., Leyda J.I., Neipp M.C., Rodríguez-Marín J. (2000). Evaluación de las dimensiones de apoyo social en pacientes oncológicos. Rev. Psicol. Soc. Apl..

[25] Rodríguez-Marín J., Terol M.C., López-Roig S., Pastor M.A. (1992). Evaluación del afrontamiento del estrés: Propiedades psicométricas del cuestionario de formas de afrontamiento de acontecimientos estresantes. Rev. Psicol. Salud..

[26] Cowling T., Jennings L.W., Goldstein R.M., Sanchez E.Q., Chinnakotla S., Klintmalm G.B., Levy M.F. (2004). Liver transplantation and health-related quality of life: Scoring differences between men and women. Liver Transplant..

[27] Ozdemir A.A., Sayin C.B., Erdal R., Ozcan C., Haberal M. (2018). Quality of Life Through Gender Role Perspective in Candidate Renal Transplant Recipients: A Report From Baskent University Using the Short Form 36 Health Survey. Exp. Clin. Transplant..

[28] Dąbrowska-Bender M., Michałowicz B., Pączek L. (2016). Assessment of the Quality of Life in Patients After Liver Transplantation as an Important Part of Treatment Results. Transplant. Proc..

[29] Bianco T., Cillo U., Amodio P., Zanus G., Salari A., Neri D., Bombonato G., Schiff S., Baggio G., Ronco C. (2013). Gender differences in the quality of life of patients with liver cirrhosis related to hepatitis C after liver transplantation. Blood Purif..

[30] Blanch J., Sureda B., Flaviá M., Marcos V., de Pablo J., De Lazzari E., Rimola A., Vargas V., Navarro V., Margarit C. (2004). Psychosocial adjustment to orthotopic liver transplantation in 266 recipients. Liver Transplant..

[31] Ministerio de Sanidad Encuesta Sobre el Alcohol y Otras Drogas en España (EDADES), 1996–2022. España: Delegación del Gobierno Para el Plan Nacional Sobre Drogas. 2023, 12–18. https://pnsd.sanidad.gob.es/profesionales/sistemasInformacion/sistemaInformacion/encuestas_EDADES.htm.

[32] Cho S., Park S., Kim J.E., Yu M.-Y., Baek S.H., Han K., Lee H., Kim D.K., Joo K.W., Kim Y.S. (2022). Incidence of depression in kidney transplant recipients in South Korea: A long-term population-based study. Sci. Rep..

[33] Craig J.A., Miner D., Remtulla T., Miller J., Zanussi L.W. (2016). Piloting a Coping Skills Group Intervention to Reduce Depression and Anxiety Symptoms in Patients Awaiting Kidney or Liver Transplant. Health Soc. Work..

[34] Heidari S., Babor T.F., De Castro P., Tort S., Curno M. (2016). Sex and Gender Equity in Research: Rationale for the SAGER guidelines and recommended use. Res. Integr. Peer Rev..

