# Supplementary material for: An Analysis of the Influence of a Patient’s Sex on Quality of Life in Liver and Kidney Transplantation
Source: Healthcare (Basel). 2024 Oct 23;12(21):2116. doi: 10.3390/healthcare12212116 (PMC11544880; doi:10.3390/healthcare12212116)
Supplement: Supplementary file 1 [file healthcare-12-02116-s001.zip › healthcare-3246055-supplementary.pdf]

Supplementary materials.

Table S1. Dates of socioeconomic status

		Liver (n=70)		Renal (n=77)	
		Men (n=58)	Women (n=12)	Men (n=46)	Women (n=31)
Marital Status	Single	7 (12,1%)	1 (8,3%)	4 (8,7%)	4 (12,9%)
	Married	40 (69,0%)	6 (50,0%)	34 (73,9%)	18 (58,1%)
	Separate	8 (13,8%)	3 (25,0%)	7 (15,2%)	6 (19,4%)
	Widower	3 (5,2%)	2 (16,7%)	1 (2,2%)	3 (9,7%)
Education	Primary	36 (62,1%)	8 (66,7%)	26 (56,5%)	15 (48,4%)
	Secondary Or Higher	22 (37,9%)	4 (33,3%)	20 (43,5%)	16 (51,6%)
Occupation	Employed/In Training Or Student	11 (19,0%)	2 (16,7%)	6 (13,0%)	5 (16,1%)
	No Current Occupation	3 (5,2%)	1 (8,3%)	3 (6,5%)	2 (6,5%)
	Home Activities	3 (5,2%)	2 (16,7%)	1 (2,2%)	6 (19,4%)
	Retired/Disability	41 (70,7%)	7 (58,3%)	36 (78,3%)	18 (58,1%)
Salary	1000€ or Less	34 (58,6%)	10 (83,3%)	19 (41,3%)	17 (54,8%)
	More than 1000€	24 (41,4%)	2 (16,7%)	27 (58,7%)	14 (45,2%)

Frequency table that details the socioeconomic status of the Transplantation list population. Subgroups with Small sample size, none of the analyzes were statistically significant.
